# Raman spectroscopy: A prospective intraoperative visualization technique for gliomas

**DOI:** 10.3389/fonc.2022.1086643

**Published:** 2023-01-05

**Authors:** Yi Zhang, Hongquan Yu, Yunqian Li, Haiyang Xu, Liu Yang, Peilin Shan, Yuejiao Du, Xiaokai Yan, Xuan Chen

**Affiliations:** Department of Neurosurgery, The First Hospital of Jilin University, Changchun, Jilin, China

**Keywords:** Raman spectroscopy, glioma, intraoperative, SERS, SRH, EOR

## Abstract

The infiltrative growth and malignant biological behavior of glioma make it one of the most challenging malignant tumors in the brain, and how to maximize the extent of resection (EOR) while minimizing the impact on normal brain tissue is the pursuit of neurosurgeons. The current intraoperative visualization assistance techniques applied in clinical practice suffer from low specificity, slow detection speed and low accuracy, while Raman spectroscopy (RS) is a novel spectroscopy technique gradually developed and applied to clinical practice in recent years, which has the advantages of being non-destructive, rapid and accurate at the same time, allowing excellent intraoperative identification of gliomas. In the present work, the latest research on Raman spectroscopy in glioma is summarized to explore the prospect of Raman spectroscopy in glioma surgery.

## 1 Introduction

Glioma is one of the most common malignant brain tumors with a high mortality rate and a low chance of cure. Currently, the treatments for gliomas consist of surgery (usually the most optimal treatment), chemotherapy (mostly alkylating agents such as temozolomide), radiation therapy, and tumor treating fields (TTFields) which is a more promising treatment modality. Over the past 20 years, neurosurgeons have constantly pursued the safety of maximizing the extent of resection (EOR) for the surgical treatment of gliomas, which has a critical impact on patient prognosis which refers to overall survival and progression-free survival etc. A growing number of studies have demonstrated the association between EOR and patient prognosis, which present that maximized EOR achieves a dramatic improvement in patient survival compared to partial resection (1–5). The infiltrative growth of glioma leads to unclear tumor boundaries and the complex relationship between tumor growth locations and important functional areas in the brain, are the most major factors affecting EOR in actual surgery for glioma, and improving the capability to recognize tumor boundaries is a relatively more plausible option. Current auxiliary methods for identifying tumor boundaries involve intraoperative fluorescence-guided microsurgery, intraoperative MRI (iMRI), intraoperative ultrasound (IOUS), intraoperative neuronavigation, and intraoperative frozen section, but there is a lack of a method that can simultaneously perform with high accuracy, rapidity, and non-invasiveness.

Raman spectroscopy (RS) is a technique that has been increasingly used in tumor detection over the past 20 years. Inelastic scattering occurs following the interaction of incident light with a material, where a small fraction of photons absorb or lose energy and change wavelength, and this process of a change in wavelength is called the Raman effect (6). Such a feature allows Raman spectroscopy to identify the chemical composition patterns of the corresponding samples. Several studies have used Raman spectroscopy in animals (7–9) and human samples (10–12) for chemical composition patterns to distinguish tumor from normal brain tissue, with excellent performance results similar to the accuracy of pathology. Moreover, the Rapidity and non-invasive properties of Raman spectroscopy also enable its potential as an intraoperative inspection method to improve the EOR of glioma surgery, so as to improve surgical outcomes and promote patient prognosis (13–15). Numerous studies have reported the applicability of Raman spectroscopy in the diagnosis of several tumors, which include colorectal cancer (16), breast cancer (17), nasopharyngeal carcinoma (18), skin cancer (19, 20), gastric cancer (21), and prostate cancer (22), etc. These studies show the prospect of Raman spectroscopy as a novel surgical adjunct to assist in the diagnosis of tumors, and the prominence of Raman spectroscopy in the diagnosis of these tumors and its specific properties - non-invasive, rapid, and accurate - makes it potentially capable of supporting neurosurgeons in the rapid identification of glioma boundaries during surgery (8, 23, 24).

The present work focuses on the advantages of Raman spectroscopy and its current applications in the diagnosis and treatment of glioma, to summarize and analyze the auxiliary role offered by Raman spectroscopy in glioma surgery. In conjunction with the recent research progress, it is expected that Raman spectroscopy might be able to provide a new perspective for neurosurgeons, that is, there is a technique that allows a surgeon to conveniently, rapidly and accurately capture the properties of tissues within the surgical field, which means that Raman spectroscopy is able to provide the power to rapidly identify tumor tissues intraoperatively so that patients can have better benefit from surgery. Meanwhile, we also integrate the latest research about Raman spectroscopy in glioma application and prospect the future development in glioma surgery.

## 2 Application of Raman spectroscopy in tumor

Raman spectroscopy, a spectroscopic technique consisting of a frequency-changing inelastic scattering caused by a change in the vibrational or rotational energy levels of molecules when a fixed monochromatic light is incident on a medium, was discovered by Indian physicist C. V. Raman in 1928, compared another one is constant frequency Rayleigh scattering caused by elastic collisions (6, 25). Raman spectroscopy was first used by Tashibu in 1990 to analyze the water content in normal and edematous brain tissue of rats by measuring the CH and OH groups (26). Subsequent studies have gradually overcome the low signal-to-noise ratio and low sensitivity of the earlier technique in recent years with the development of optical and data processing techniques, and studies using the technique to identify tumor cells on postoperative Formalin-fixed paraffin-embedded (FFPE) pathology sections have gradually emerged (15, 27). Latest studies have also utilized intraoperative frozen pathology sections (14), intraoperative fresh tissue blocks (28), and *in situ* tissue in the operative area (11, 29) for the identification and diagnosis of gliomas, while data processing techniques such as machine learning and deep learning have also been used to enhance the accuracy of Raman techniques (30–32).

**Figure 1:**
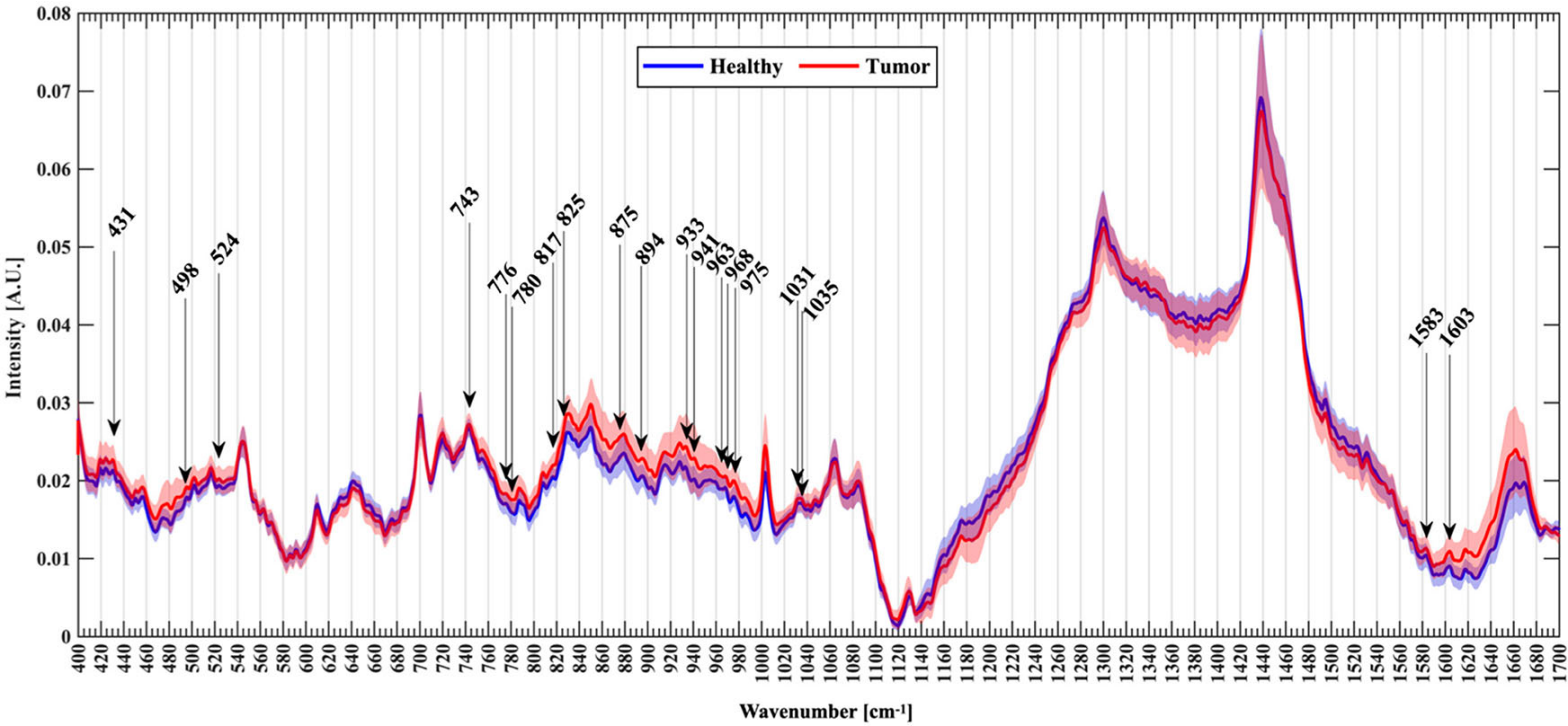
An example of Raman spectroscopy shows normalized mean spectra with standard deviation between healthy (blue) and tumor patients (red). Arrows mark the new Raman peaks about glioma identified by the researcher (28).

Since the composition of nucleic acids, proteins and lipids in tumor cells are dramatically different from normal brain cells, while Raman spectroscopy is able to identify the chemical composition of a sample by measuring the Raman shift caused by the difference between the frequency of the scattered light and incident light in the Raman effect (33), as Ji reported that glioma and normal brain tissues have significantly different spectral peaks at 1080 cm-1 (nucleic acid), 2845 cm-1 (lipid) and 2930 cm-1 (protein) positions (34). Instead, the differences just depend on their chemical group, independent of the excitation wavelength, while the intensity of Raman peaks is related to the excitation light wavelength, power and concentration of the measured substance (35), so the Raman spectroscopy have the possibility to evaluate tumors and normal tissues according to the differences (**Figure 1**) (12). Currently, the major diagnostic methods for glioma in clinical practice include imaging and pathology, and imaging primarily covers CT, MRI and PET. CT serves merely as a way to screen for tumors, whereas MRI is a more reliable way to assess the properties of tumors. MRI is based on the nuclear magnetic resonance (NMR), according to the different attenuation of the energy released in different structural environments within the material, the position and type of atomic nuclei of the object can be learned through the detection of the emitted electromagnetic waves by the applied gradient magnetic field, and the internal structure of the object can be visualized accordingly (36), nevertheless, MRI is an indirect method to determine the morphology and properties of tumors, without a satisfactory sensitivity and specificity. PET, a new imaging method that has developed rapidly in recent years, labels the tumor-specific metabolic substances with radioisotopes to judge the properties and morphology of tumors *via* the metabolic characteristics of intracranial occurrences by using the metabolic characteristics of tumors. The current gold standard pathology for tumor diagnosis consists of formalin fixation of intraoperatively resected tissues, paraffin embedding and staining of sections, followed by microscopic view of cellular staining, morphology, size, arrangement pattern and other cytological features to make a diagnosis, which relies on the subjective judgment of experienced senior pathologists. Consequently, the complexity of the process and the requirement for experienced pathologists contributes to a time-consuming approach to pathology diagnosis; additionally, biomarkers are typically detected in other tumors, which are not as widely available in gliomas due to factors like the blood-brain barrier. The high sensitivity of Raman spectroscopy to the detected substances provides accurate results, and the detection time of only tens of seconds greatly boosts the efficiency of detection.

Despite the high sensitivity and accuracy of Raman spectroscopy, the Raman signal received by the device is remarkably weak since only one ten-millionth of the photons will produce the Raman effect. Meanwhile, a lot of factors in the environment will influence the final acquisition result, thus various Raman signal enhancement methods have been developed, such as Resonance Raman spectroscopy (RRS), surface-enhanced Raman spectroscopy (SERS), tip-enhanced Raman spectroscopy (TERS) and stimulated Raman spectroscopy (SRS) etc. (35) One of the most sensitive detection modalities is SERS, which can enhance the Raman signal by 10^6 - 10^14 times and increase the detection level to single molecules, except that a metal surface or metal nanoparticle surface is required (37, 38). Other Raman imaging techniques useful for intraoperative glioma surgery are coherent anti-Stokes Raman scattering (CARS) microscopy and stimulated Raman histology (SRH). CARS and SRS are coherent Raman imaging that coherently affects the Raman effect of a specific chemical bond by emitting a second light source to increase the signal intensity. SRH is the only Raman spectroscopy method approved by the FDA for now, which uses SRS to generate virtual imaging of fresh tissue whose imaging quality approximates H&E section images (39). Raman shift is the part of the Raman effect that varies in frequency, and whose common range is 400-3500 cm-1. The results of Raman spectroscopy can be divided into the Raman fingerprint region (FP, 400-1800 cm-1), the high wavenumber region (HW, 2,800-3,200 cm-1), and the intermediate region according to wavenumber. In most studies, simultaneous acquisition of the FP and HW regions has shown a higher value, which means the Raman results should be analyzed focusing on the 400-1800 and 2800-3200 cm-1 (40, 41), and should increase the integration time as much as possible instead of increasing the laser power (42).

Raman spectroscopy plays a role in the detection of many cancers, and some studies have exploited the high sensitivity of Raman spectroscopy to chemical components to detect colorectal cancer through liquid biopsies and endoscopy with direct access to the lesion (16). As in gastric cancer, which is similar to colorectal cancer, studies have applied Raman spectroscopy to diagnose gastric cancer through tissue removed during surgery or endoscopic biopsy, besides being able to analyze patients with gastric cancer *in situ* in real time using fiber optic probes (21, 35). Raman spectroscopy in breast cancer is dominantly performed from breast samples, including handheld Raman probes for tissue classification and assessment of surgical margins (17). Since skin cancer lesions usually lie on the surface of the body and facilitate Raman spectroscopy, several studies examined skin cancer biopsy specimens for Raman detection to improve the time and convenience needed to confirm the properties of the excised tissue intraoperatively, as well as the strict requirement to expand the extent of excised tumor for reasons similar to the aesthetic needs arising from the excision of facial tumors, where Raman spectroscopy is used to confirm the properties of the edge of the surgical resection area as much as possible intraoperatively (19, 20). The successful application of Raman spectroscopy in tumors mentioned above and others for instance nasopharyngeal carcinoma (18) and prostate cancer (22) reveals that Raman spectroscopy accepted a wide range of biological samples thanks to its highly sensitive features than the other detection means. The applications of Raman spectroscopy in the diagnosis and treatment of glioma include tumor tissue (43), in which typically tumor cells have higher protein and less lipid, and the fingerprint profile of chemicals that are different for tumor and normal tissues can be targeted to distinguish the properties of a given tissue. Brain-tumor margin tissue can be depicted by SRH, CARS and other methods as a virtual image, and then delineate the tumor margin at the cellular level by pre-trained predictive models (44). As a result of the presence of altered microenvironment around the tumor, Raman can also indirectly identify tumors based on the changes in the chemical composition of the body fluids, e.g., the pH value is generally lower in the microenvironment around the tumor (29, 45). The blood of tumor patients commonly exhibits elements from tumors like exosomes, and Raman provides an indirect indication of the presence of tumors by working with it (46). *In-situ* tissue in the operative area (11), which is one of the most prospective developments in surgery, Raman directly measures the molecular composition signatures of the *in-situ* tissue in the operative area by fiber optic probe, then offer an answer for tumor or not through pre-trained prediction algorithms, providing the operator with a decision aid on whether or not the tissue of the operative area should be extended for resection during the surgery.

## 3 Raman spectroscopy for glioma resection

The most common adjuncts applied in glioma surgery today range from intraoperative fluorescence-guided microsurgery, intraoperative MRI (iMRI), intraoperative ultrasound (IOUS), intraoperative neuronavigation, and intraoperative frozen section. Intraoperative MRI is a technique that has been developed over the past 20 years to evaluate the EOR during surgery by using the MRI machine installed in the operating room after eliminating factors that may affect the MRI machine in the operating room (47), but it must be designed before the construction of the operating room in order to install perfectly the intraoperative MRI, besides, the time and environmental requirements of the examination have limited the popularity of this technique. More rapid depiction of Raman spectroscopy in the operative area versus intraoperative MRI allows results to be reported within tens of seconds, and Raman detection equipment has better mobility than MRI equipment, requiring less environmental requirements for use without special operating room planning before. Intraoperative ultrasound is considered a simple and inexpensive surgical adjunct, as it is a cheaper device than MRI and the probe can be deployed intraoperatively, and the detection time is instant, but limitations of the ultrasound technology itself mean that its sensitivity for residual tumor identification is poor and dependent on an experienced surgeon. With relatively inexpensive detection equipment and slightly slower detection speed than ultrasound, Raman spectroscopy has superior sensitivity and specificity for tumor tissue identification. Intraoperative neuronavigation is usually performed by preoperative high-resolution MRI, and the patient’s head is fixed relative to the navigation markers in preoperative preparation, and then the exact position of the probe in the patient’s head is displayed on the screen in real-time by locating the probe position intraoperatively after position information is registered. Intraoperative navigation offers high accuracy and timely feedback on the location of the probe, but the cerebrospinal fluid released during brain surgery will result in the relative position of the brain tissue to the registration point changes or drifts, which leads to the position indicated by the intraoperative navigation no longer be accurate and thus the relationship between the location of the probe and the tumor can no longer be determined. Raman spectroscopy does not be affected by the intraoperative drift problem as the samples located *in situ* tissues or removed from the tissues of operative area intraoperatively. Intraoperative fluorescence-guided microsurgery aims to indicate the location of the tumor intraoperatively by injecting fluorescent contrast agents such as 5-aminolevulinic acid (5-ALA) or fluorescein sodium into the patient intraoperatively or preoperatively, taking advantage of the fact that glioma would disrupt the blood-brain barrier and therefore induces the fluorescent agent to be trapped and accumulated in the tumor. In contrast, Raman spectroscopy is a label-free method that does not require the addition of drugs to label the tumor. Although the intraoperative frozen section is an intraoperative adjunct providing the pathology of the excised tissues to the surgeon, due to the high water content of the brain tissue and the softness of the tissue, the Intraoperative frozen section for gliomas is frequently confused by the morphology of the tissues, which prevents the pathologist from reaching a more valid conclusion. With Raman spectroscopy, it is possible to achieve intraoperative results in just a few tens of seconds, while keeping the accuracy close to that of postoperative FFPE pathology (11, 48–50). In summary, Raman spectroscopy as an intraoperative technique has the great advantages of being more rapid, label-free, accurate, and non-invasive than current intraoperative diagnostic methods.

### 3.1 Raman spectroscopy for intraoperative fresh tissue determination of glioma

**Figure 2:**
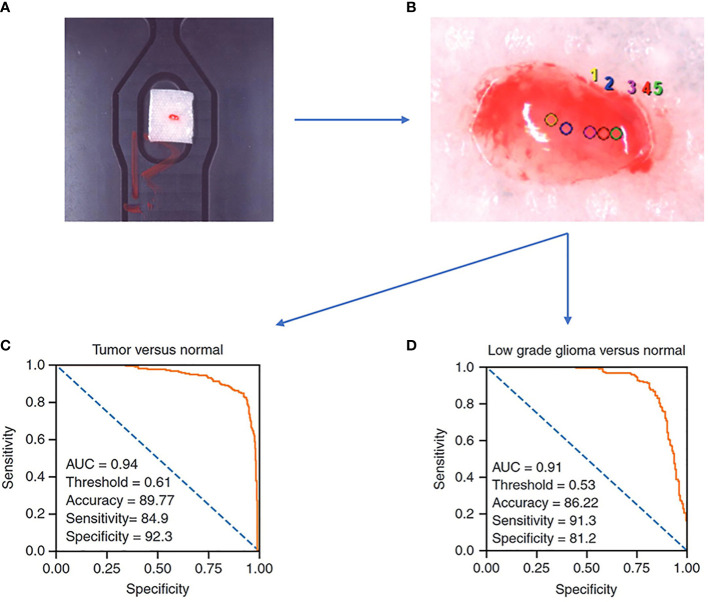
A Raman spectroscopy study examines freshly collected ex vivo brain tissue and achieves good results with a predictive model constructed by machine learning algorithms. **(A)** samples of size 2-4 mm in length, width, and height are needed. **(B)** Selection of sites for Raman spectroscopy. **(C)** ROC curve of a trained logistic regression model to identify tumor and normal brain tissue. **(D)** ROC curve of a trained logistic regression model to identify low grade glioma and normal brain tissue (30).

One of the most common and direct applications of Raman spectroscopy is to examine the intraoperatively obtained tissue directly, and the results derived in this way represent the actual intraoperative situation of the tissue more directly and better than FFPE samples. A prospective study in 2022 carried out direct Raman imaging on 29 freshly collected ex vivo brain tissue samples, each of which was split into 2-4 mm and examined for pathology, and finally, the results were trained by machine learning algorithms as a predictive model that could classify tumors from normal tissue with an accuracy of 89.8%, sensitivity of 84.9%, and specificity of 92.3%, as well as LGG and normal tissue with an accuracy of 86.2%, sensitivity of 91.3%, and specificity of 81.2% (**Figure 2**) (30). By analyzing a total of 3450 spectral results from 63 fresh glioma samples, Riva et al. identified 19 Raman shifts specific to glioma, whereby the predictions could reach 82% precision (28). A study has proposed that the effect of Raman spectroscopy is superior to the value derived from 5-ALA, while Livermore’s team created a model by samples from actual patients following a PCA-LDA machine learning approach and compared the predictive effect of 23 samples taken from 8 patients during surgery with 5-ALA, where each sample was confirmed under the microscope to confirm 5-ALA, showing that Raman spectroscopy (1.00 sensitivity, 1.00 specificity, and 1.00 accuracy) was significantly better than 5-ALA (0.07 sensitivity, 1.00 specificity, and 0.24 accuracy (p = 0.0009)) for prediction of glioma (27).

From the above study, it is clear that the power of Raman spectroscopy to differentiate glioma from normal brain tissue is robust when augmented by simple machine learning algorithms compared to current intraoperative adjuncts like intraoperative fluorescence-guided microsurgery. According to the high precision identification of glioma by Raman spectroscopy, surgeons can obtain the properties of the intraoperative excised tissue by subjecting it to rapid inspection to determine whether the tissue at the current point of interest in the operative area is tumor or normal tissue for further surgical decisions. Raman spectroscopy of intraoperatively extracted tissues requires minimal operator effort, has little impact on the present surgical procedure. Simultaneously, RS is a more preferable way of detection with fresh tissue retaining the most original information. But correspondingly, the Raman detection which is usually to predict the type of tissue extracted by pre-training the model yields limited results and lacks more information to make judgments such as virtual staining.

### 3.2 Stimulated Raman histology for rapid intraoperative diagnosis of gliomas

**Figure 3:**
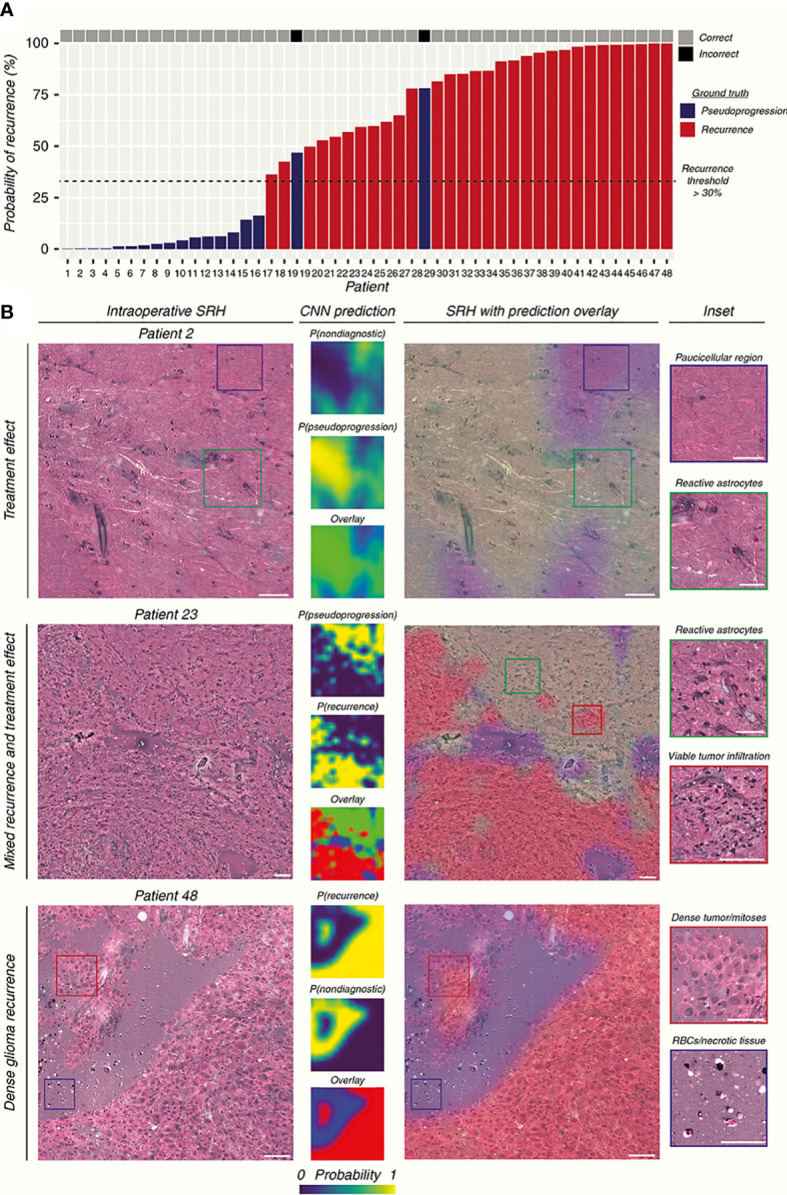
The researchers used CNN to construct the prediction model by SRH images. **(A)** The prediction results of the SRH-based prediction model are demonstrated. **(B)** SRH images and CNN probability heatmaps. The model was able to correctly identify pseudoprogression, tumor recurrence, and infiltrative glioma. Scale bars = 50 μm (52).

Typically, the Raman effect is so weak that the detection equipment is often subject to various external disturbances. Coherent Raman spectroscopy was born as an attempt to enhance the Raman effect, which affects the Raman effect of a specific chemical bond by a second incident beam of light coherently, increasing the signal intensity. SRH builds on the technology of Raman spectroscopy to generate virtual tissue imaging of slices so that the results of Raman spectroscopy are no longer limited to just being a classifier, instead, providing more information such as the cellular morphology, arrangement, and structure of the tissue. Hollon’s team demonstrated in a large sample multicenter, prospective clinical trial (n = 278) that the diagnostic accuracy of SRH-based images is consistent with that of pathologists for conventional histological images (overall accuracy, 94.6% vs. 93.9%) (51). After training on 2.5 million SRH images using convolutional neural networks (CNN), a neural network structure widely used in image recognition and inspection, the team applied the model in the operating room to predict brain tumors, and the model could distinguish not only tumor tissue with high accuracy but even the main histopathological classification of brain tumors, in addition to identifying tumor infiltrates from SRH images. In a later study, Hollon’s team enhanced the SRH technique with fiber optic laser imaging in 35 patients with recurrent glioma, after which the resulting SRH images were trained using a CNN, and finally, the trained model was applied to a retrospective cohort to score a diagnostic accuracy of 95.8% (**Figure 3**) (52). This demonstrates how more advanced algorithms combined with more advanced Raman imaging techniques can play a significant role in the diagnosis of gliomas, not only by achieving intraoperative accuracy similar to that of conventional pathological diagnosis but also by producing results in an order of magnitude faster than intraoperative frozen section, making it more promising as a novel method that can join or even replace existing intraoperative assist techniques. Another study conducted a blinded, prospective cohort study in 21 patients with central nervous system (CNS) tumors, investigating the differences in accuracy and diagnostic time between conventional pathology and Raman spectroscopy, found no significant differences in diagnostic accuracy between the methods (P = 1.00) and a significantly shorter mean time to diagnosis (TTD) for SRH-based diagnosis than frozen sections (43 min versus 9.7 min, P < 0.0001) (12). This study illustrates that Raman spectroscopy reduces the time required for diagnosis significantly while maintaining a high level of accuracy, and has great advantages in neurosurgery, by comparing the SRH-based diagnostic method with the traditional gold standard diagnostic method of frozen sections and FFPE sections.

The breakthrough of SRH in glioma surgery is far more exciting, as it broadens the use of Raman spectroscopy, enabling RS not only to distinguish tumor tissue from normal tissue but also to present Raman-detected results in the form of images, not only to overcome some of the shortcomings encountered in the intraoperative frozen section but also more fully exploit the characteristics of Raman spectroscopy for the detection of samples with high sensitivity, which enables pathologists to open the third eye in the existing diagnostic process of the intraoperative frozen section, thus improving the diagnostic ability and further promoting the EOR of glioma surgery. This approach provides the surgeon with more accurate and detailed visualization of the tumor boundary than the Section 3.1 approach. However, SRH still requires further processing of the sample and cannot immediately image the tissue removed intraoperatively, so its usefulness remains to be explored.

### 3.3 More convenient handheld Raman spectroscopy

**Figure 4:**
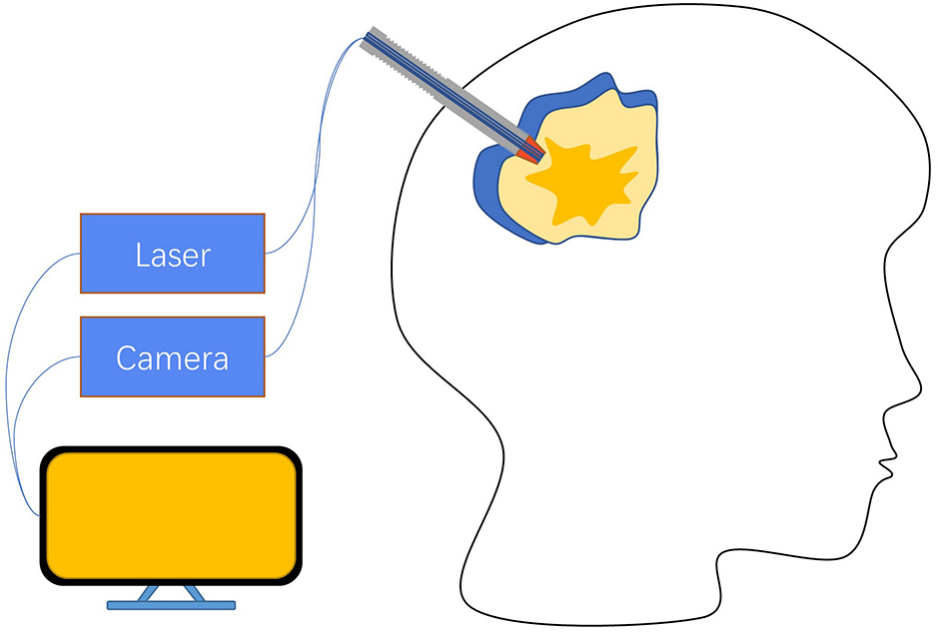
A hand-held fiber optic probe consists of a laser emitter connected to a spectral detector, with data acquisition ultimately controlled by a PC. The illumination and detection light paths are spatially coincident. When the probe performs measurements, it is in direct contact with the brain at the resection edge (53).

Because of the optical path design and other reasons in Raman spectroscopy equipment, most detection devices are designed to be larger, and the detection method also usually requires a series of steps that the samples need to be placed on the detection table. Although such complex operation logic ensures that it will not affect the patient itself, it still requires removing the tissue from the surgery and trimming it to the right size before putting it into the device for detection, which invariably prolongs the detection time and may lead to the risk of removing more normal tissues caused by edge uncertainty. These issues in the research process will probably not pose a serious impact, but if Raman technology is expected to become clinical tool, it must possess features that can be easily operated by neurosurgeons. Based on the optical properties of Raman spectroscopy, some works have been done to solve the above problems of complex operation logic by designing fiber optic probes, and some practical applications have been made. Evaluation of the feasibility and accuracy of handheld Raman spectroscopy devices as an intraoperative neuronavigation adjunct was performed on a dog model (9). Eleven tumor resection and intraoperative handheld Raman devices were examined on 11 dog models, and Raman signals were collected using the handheld devices in direct contact with the intraoperative tissue, and pathology was determined for the sample sites, finally, the results showed that the handheld device had a sensitivity of 85.7% and specificity of 90% with a positive predictive value of 92.3% and negative predictive value of 81.6% compared to pathology. These efforts validate the feasibility of using handheld devices in neurosurgery by providing rapid intraoperative results for the operator’s reference with comparable accuracy to gold standard pathology. Apart from that, it would be a great help for surgery if the tumor border location could be provided to the operator intraoperatively through a handheld device. A handheld macro Raman imaging system was reported to be designed for detecting tissue edge features, and the team concluded that it is necessary to work on a detection system with a larger field of view for tumor margin detection in hand (23). In this project, they conducted experiments on the fat and muscle tissues of pigs and trained the classifier with the obtained data using machine learning methods, which resulted in an *in vitro* validation result of 99% accuracy and plotting the boundary probabilities by the phenomenon that the classifier is found to decrease the prediction rate at the junction of fat and muscle tissues. What’s more interesting is that a study found that tumor infiltration was still detected 3.7-2.4 cm outside the MRI-determined tumor border with 92% accuracy, 92% sensitivity, and 93% specificity (53), using a fiber-optic probe in direct contact with the brain at the edge of the resection cavity in surgery with a handheld Raman imaging system during a total acquisition time of 0.2 seconds per measurement (**Figure 4**). The results were evaluated by H&E section pathology, and the effect of the handheld RS device was undoubted to improve the surgical outcome and prolong patient survival.

Compared to the Raman spectroscopy method described in Section 3.1, there is no visible difference in the accuracy of the handheld Raman spectroscopy method, but the handheld Raman spectroscopy device significantly improves the convenience for surgeons to use during surgery. These portable handheld Raman spectroscopy systems address the complexities of previous studies that require sample isolation and avoid patient harm. These portable handheld Raman spectroscopy systems address the complexities of previous studies that require sample isolation and avoid patient harm. Combined with the relatively small size of the device, fast acquisition time, and high accuracy, RS is a convenient way for neurosurgeons to detect tissue properties in the operative area, identify tumor cell margins and infiltrations, provide guidance for surgical procedures, and improve EOR compared to other intraoperative detection methods, and has great potential to serve as a new method to supplement or even replace existing intraoperative tumor margin detection methods. Unfortunately, the interaction between the incident laser and brain tissue needs to be taken into account when handheld Raman devices are used intraoperatively to assist the operator in analyzing the properties of the resected sample. Generally, Raman spectroscopy is considered to be harmless to humans, but the potential long-term damage to brain tissue from repeated applications over a long period of time is not known, so the safety and necessity of RS before widespread use in the clinic needs to be further explored (17).

### 3.4 Unprecedented rapid intraoperative molecular detection

**Figure 5:**
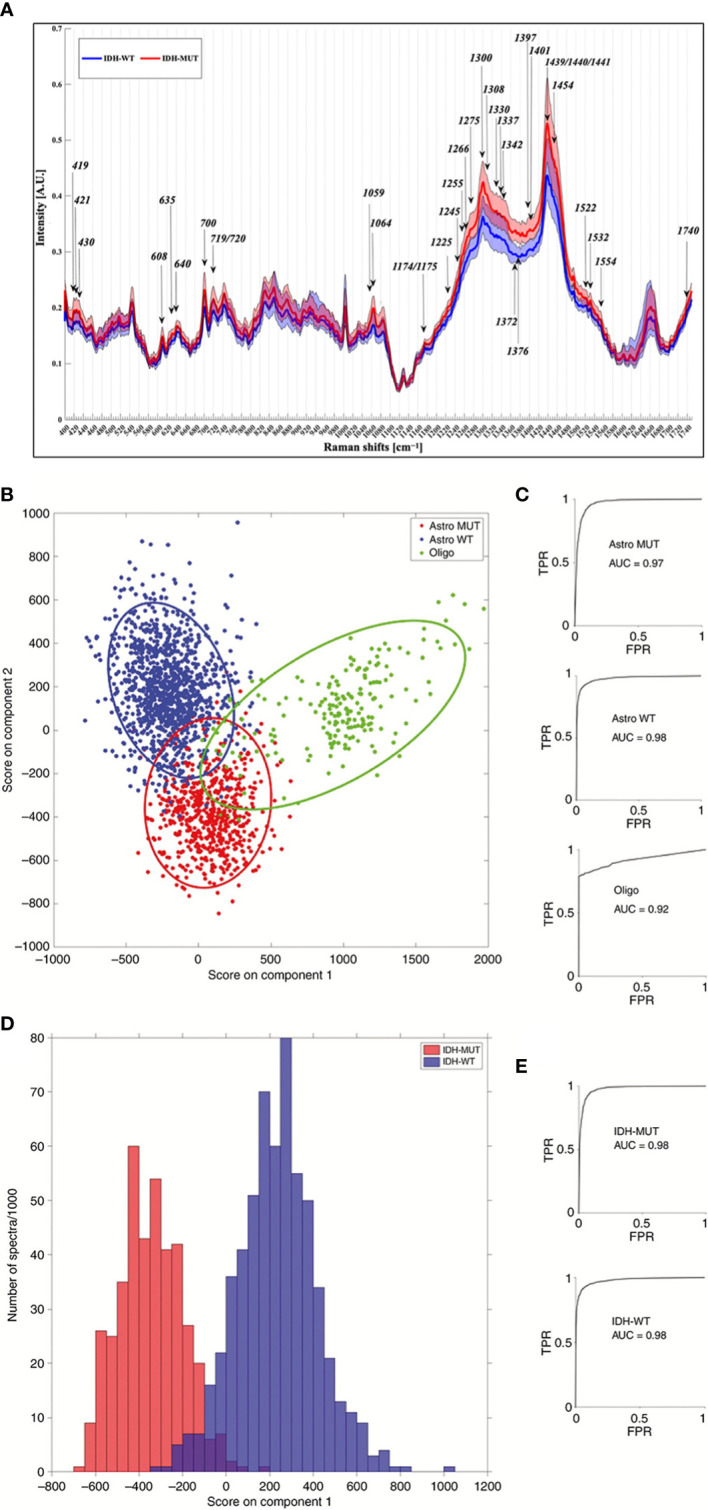
**(A)** Results of one study for IDH-WT and IDH-MUT, with arrows representing the most discriminant peaks with a known biological assignment. **(B–E)** are the 3-group model and 2-group model, respectively, of another study for molecular typing of gliomas based on Raman spectroscopy results. (TPR = true positive rate; FPR = false positive rate; Astro MUT = Astroglial tumor isocitrate dehydrogenase IDH-mutant; Astro WT = Astroglial tumor, IDH-wild-type; Oligo = Oligodendroglioma) (14, 55).

Based on the 2016 WHO classification system of the CNS, glioma could be classified as followed: lower grade glioma (LGG) with isocitrate dehydrogenase (IDH) mutation with or without 1p/19q-codel, LGG with IDH-wildtype subtype, glioblastoma multiforme (GBM) with IDH mutation or not (54). IDH and other molecular biomarkers have become a critical part of the glioma diagnosis and treatment process. As Raman spectroscopy is excellent in discriminating substances, a few works have been conducted on the ability of Raman spectroscopy to distinguish IDH subtypes in glioma biopsies with 2073 Raman spectroscopic results taken from 38 samples, using the eXtreme Gradient Boosted trees (XGB) and Support Vector Machine with Radial Basis Function kernel (RBF-SVM) to perform the classification task and found 52 different Raman shifts between IDH-mut and IDH-wt groupings, which shifts representing lipids, collagen, DNA and cholesterol/phospholipids, with a final accuracy of 87% (14). Further studies have predicted glioma IDH subtypes by a simple PCA-LDA method on fresh tissue samples from 62 patients, achieving 91% sensitivity and 95% specificity (**Figure 5**) (55). Moreover, subtype differentiation by differences in protein profiles (498, 826, 1003, 1174, and 1337 cm-1 were selected) between different molecular subtypes of glioma (n = 36) was also studied with a likewise high accuracy rate of 89% (43).

Given the high sensitivity of Raman spectroscopy for the detection of samples, Raman spectroscopy is playing an increasing role in the diagnosis and treatment of glioma in the future as molecular biology becomes a growing involvement in glioma. The ability of Raman spectroscopy to rapidly assess the molecular biology of glioma samples during surgery provides the operator with a three-dimensional understanding of the possible grading and staging of glioma, and then adjusts the EOR according to the degree of malignancy to achieve a better treatment prognosis. The application of Raman spectroscopy in glioma surgery is a unique approach to intraoperative adjuncts commonly used in clinical practice, as it can provide the operator with long postoperative molecular typing detection in near real-time, which is a novel approach for gliomas whose malignancy cannot be easily identified intraoperatively.

## 4 The development direction of Raman spectroscopy

Raman spectroscopy can be safely used in clinical practice owing to the fast, accurate, non-invasive, and label-free features that enable it to be performed without more interaction with the patient’s body. Yet, the Raman effect is very weak considering that the photons producing the Raman effect are merely one ten-millionth of the incident light, and the weak interference in the environment will affect the final imaging results. Coherent Raman techniques such as SRS and CARS can significantly increase the Raman signal intensity, but a more sensitive SERS technique can amplify the Raman effect by 10^6-10^14 times through a metal surface, even to the resolution of single molecules (56). Besides, the huge amount of data generated per second by the high resolution of Raman spectroscopy render more advanced data processing techniques necessary. Nowadays, concerning the results of Raman detection, except for the traditional statistical methods such as principal component analysis (PCA), partial least squares (PLS), linear discriminant analysis (LDA), etc. to find the differences between glioma and normal brain tissue (27, 31, 51, 55), machine learning algorithms such as k-nearest neighbors (KNN), support vector machine (SVM), random forest (RF) and eXtreme Gradient Boosting (XGB) are quite often adopted to perform classification and prediction (14, 57–59). With the hot trend of neural network algorithms over the past few years, a growing number of deep learning algorithms have been trained to predict Raman detection results, commonly available methods include convolutional neural networks (CNN), artificial neural networks (ANN), etc. (32, 52) While hot new frameworks such as long short-term memory (LSTM) and Transformer (60, 61) also have application potential in targeting Raman detection results, the new algorithms can be expected to give us more surprises in predicting.

### 4.1 Ultra-high sensitivity Raman spectroscopy method brings new visions

SERS was developed to enhance the Raman scattering of molecules from nanostructured materials, allowing the detection of very low levels of material, even individual molecules, through a proton-mediated enhancement effect (56), and the extremely high sensitivity making SERS important for the intraoperative detection of gliomas. A SERS probe with a detection limit of 5 pM in an aqueous solution was reported to be developed to delineate tumor invasion margins by a time-distance function on a mouse glioma xenograft model using extravasation in the tumor vasculature of the probe, and resection experiments were performed with the aid of handheld Raman scanning equipment (8). The experimental procedure resembled intraoperative fluorescence-guided surgery, which ultimately improved the overall survival rate of the rat model compared to normal resection and possessed a detection accuracy of pM precision than fluorescent methods such as 5-ALA. The Warburg effect refers to the tendency of tumors to metabolize anaerobically, which is reflected in the large amount of lactic acid produced by tumors during the metabolic process (62). The SERS technique has been used to define tumor boundaries by preparing a SERS substrate 4-mercaptopyridine (4-MPY) to detect changes in pH around the target based on the Warburg effect, which introduces gliomas produce large amounts of lactic acid that cause their microenvironmental pH to drop and appear acidic, and 4-MPY is a type of silver nanoparticle with different SERS peak characteristics at different pH values (29). In follow-up work, the team did the same thing using U87 cells grown in mice. Building on the same characterization of the tumor microenvironment as acidic, a study was conducted in animal experiments to develop a SERS-based surgical navigation system to identify tumor boundaries, which was detected by transferring metabolites from tumor resection margins to a PH-sensitive SERS chip (45). According to the research, the overall survival of animal models operated under this surgical navigation system was dramatically increased, but unfortunately, exogenous probes were not used in the study due to approval issues by the drug supervision authority (45).

The ultra-sensitivity of SERS permits the detection of gliomas from a wider range of angles, such as more accurate biomarker results for intraoperative samples of gliomas. Whereas the need to prepare nano-metals limits its use in humans, we can still access patient samples from other angles, such as blood (63) to evaluate the postoperative outcome of glioma patients, as well as the routine screening of normal people. Because of the SERS technology advances and the indications for nanometals in humans continue to expand over time, it is expected that SERS will accomplish greater improvements in intraoperative glioma guidance.

### 4.2 Raman spectroscopy desires a better data processing method

Whether using Raman spectroscopy as a method of glioma identification or intraoperative generation of virtual tissue images, or as an intraoperative aid to the operator, one needs to face the large amount of data generated by Raman spectroscopy, and how to use these Raman data to get better-expected results is a part that needs attention. Generally speaking, Raman spectroscopy results are obtained in two forms, one is the result of molecular composition information represented by Raman shift raw data, and the other is the result of virtual images generated by SRH, CARS, etc. In recent studies, the most popular way of analyzing the data collected by Raman spectroscopy in glioma continues to be the dimensionality reduction approach with PCA, PLS, and LDA (27, 31, 51, 55). Taking the data collected by Raman spectroscopy and extracting the main differences by which tumors and normal cells can be distinguished, these studies often achieve excellent accurateness thanks to the inherent high sensitivity of Raman spectroscopy. In order to further improve the accuracy of Raman spectroscopy and make the new technology more acceptable to clinicians, a number of studies set out to process the data from Raman spectroscopy using popular machine learning methods, and some currently growing neural network algorithms have been added to the processing of Raman spectroscopy results. Some scholars reduced the dimensionality of the data by PLS and PCA, after which the principal components were selected using four methods, Relief-F, Pearson correlation coefficient (PCC), F-score (FS) and term variance (TV), and finally, back propagation neural network (BP), linear discriminant analysis (LDA) and support vector machine (SVM) classification models were established (58); Sciortino et al. used eXtreme Gradient Boosted trees (XGB) and a support vector machine with Radial Basis Function kernel (RBF-SVM) to evaluate classification performance (14, 59), with a LOO approach to achieve a balanced trade-off between performance and robustness; Stables employed three methods, SVM, KNN and LDA, to classify glioma samples (57); Another scholar has focused on feature engineering to develop a new representation specifically for brain diagnosis while retaining as much information as possible to improve prediction accuracy (31); Jermyn argues that ANNs can overcome the aspect of spectral artifacts generated by lights in operating rooms using nonparametric and adaptive models, reducing the changes required in neurosurgical workflows for Raman spectroscopy detection thereby simplifying the barriers to intraoperative use of Raman spectroscopy (32); CNNs, which excelled in the image field, were also trained to recognize SRH images to detect recurrent gliomas (52); and deep learning models based on simulated annealing algorithms were also applied to deal with the Raman spectroscopy detection results of gliomas (64).

All these efforts have revealed enormous value in the study of Raman spectroscopy of glioma, effectively boosting the predictive ability of Raman spectroscopy for glioma, creating a new direction for future efforts to improve the application of Raman spectroscopy, and enhancing the value of Raman spectroscopy applications. However, it is undesirable to create excessive reliance on algorithms and attempt to take care of all the problems of inaccurate results arising from external factors such as sampling, environment, and improper usage through algorithms. A proper approach should be to ensure the accuracy and standardization of each step in the data acquisition process as much as possible, minimize the interference brought by the outside world, and finally improve the robustness by multiple sampling or upgrading the algorithm. Thanks to the rapid development of deep learning in recent years, more algorithms can be applied to the processing of Raman spectroscopy in the future, such as Transformer and LSTM, both of which do not seem to have been applied to the data processing of Raman spectroscopy in glioma (60, 61), the potential of the algorithm in improving the accuracy of Raman spectroscopy detection is still wide.

## 5 Conclusion and future perspectives

Today, various clinical techniques are available to assist in the EOR of glioma surgery, but they are limited in terms of invasiveness, speed of detection, and accuracy, which slow down the improvement in the EOR of glioma surgery. Raman spectroscopy, however, has excellent characteristics that make it possible to identify tumor margins in glioma surgery while being non-invasive, fast, and accurate. Raman spectroscopy not only compensates for the shortcomings of current intraoperative adjuncts but also enables the detection and understanding of glioma at the molecular level, while the research of fiber optic probes also gives Raman spectroscopy a broader scope of application. But some problems still exist in the application of Raman spectroscopy in glioma, firstly, the sample acquisition, most of the Raman detection devices need to transfer the intraoperative tissues to the detection devices for detection, and SRH technology needs to detect and image the slices. Secondly, Raman spectroscopy results cover a large amount of raw data, and it is still a proposition worth exploring how to extract the key from the huge amount of data and how to enhance the data processing algorithm to improve the accuracy. Additionally, the Raman effect requires a certain power of incident light as the excitation, and SERS and other Raman techniques demand the assistance of nanomaterials, all of which need to be in contact with the patient’s brain tissue, and the potential safety hazards brought by these operations should be strictly evaluated to avoid secondary injuries to patients. Precisely speaking, Raman spectroscopy has a most attractive prospect in glioma surgery, and there is a steady stream of research on the application of Raman spectroscopy in glioma, suggesting that Raman spectroscopy will one day become the most commonly used intraoperative adjunct technique in clinical practice.

## Author contributions

YZ and HY contributed equally to write the original manuscript and should be considered co-first authors. YL and HX provided suggestions for revisions. LY, PS, YD and XY provided pictures and critical revisions. XC reviewed and edited the manuscript. All authors contributed to the article and approved the submitted version.
